# Echocardiogram-guided resuscitation versus early goal-directed therapy in the treatment of septic shock: a randomized, controlled, feasibility trial

**DOI:** 10.1186/s40560-018-0319-3

**Published:** 2018-08-13

**Authors:** Michael J. Lanspa, Rebecca E. Burk, Emily L. Wilson, Eliotte L. Hirshberg, Colin K. Grissom, Samuel M. Brown

**Affiliations:** 1 Critical Care Echocardiography Service, Intermountain Medical Center, 5121 South Cottonwood Street, Murray, UT 84107 USA; 2 Division of Pulmonary and Critical Care Medicine, University of Utah School of Medicine, Salt Lake City, UT 84132 USA; 3 Division of Critical Care, Department of Pediatrics, University of Utah School of Medicine, Salt Lake City, UT 84132 USA

**Keywords:** Septic shock, Sepsis, Fluid therapy, Echocardiography, Early goal-directed therapy, Randomized controlled trial

## Abstract

**Objective:**

Echocardiography is often used to guide septic shock resuscitation, but without evidence for efficacy. We conducted an intensive care unit (ICU)-based randomized controlled feasibility trial comparing echocardiography-guided septic shock resuscitation (ECHO) with early goal-directed therapy (EGDT).

**Methods:**

We conducted a single center, randomized controlled feasibility trial at a 468-bed academic tertiary care center in Utah, USA. Adult patients with early septic shock were assessed and treated at defined intervals over 6 h using an echocardiogram-guided resuscitation protocol or a slightly modified EGDT protocol. Feasibility outcomes were fluid balance, dobutamine administration, and time to lactate clearance. The primary clinical outcome was changed in sequential organ failure assessment score at 48 h (delta SOFA). Secondary outcomes included inpatient mortality, ICU-free days, and ventilator-free days at 28 days.

**Results:**

Thirty participants, 15 per group, were randomized and completed the study. Baseline characteristics were similar between groups. Patients were randomized within a median of 3.5 h of meeting inclusion criteria but had received a median of 3 L crystalloid by then. Fluid administration during the study protocol was similar in both groups (median ECHO 0 vs EGDT 1 L, *p* = 0.61). Eleven (73%) subjects in each arm received ≤ 1 L fluid. Dobutamine administration was also similar (20% vs 13%, *p* > 0.99). Twenty-one patients (70%) had lactate clearance prior to the first study assessment. No difference was observed in delta SOFA (median − 4 for ECHO vs − 6 for EGDT, *p* = 0.10) nor mortality (33% ECHO vs 20% EGDT, *p* = 0.68).

**Conclusions:**

No experimental separation was observed in this randomized, controlled feasibility trial. Early lactate clearance, coupled with substantial fluid administration before randomization, suggests that patients were already resuscitated before arrival in the ICU. Future trials of echocardiogram-guided sepsis resuscitation will likely need to enroll in the emergency department.

**Trial registration:**

This study was retrospectively registered at clinicaltrials.gov (identifier NCT02354742, title Echo vs EGDT in severe sepsis and septic shock) on February 3, 2015. Registration was completed before review or analysis of any data.

**Electronic supplementary material:**

The online version of this article (10.1186/s40560-018-0319-3) contains supplementary material, which is available to authorized users.

## Background

Septic shock is a common cause of death, with current mortality rates between 20 and 40% in resource-rich settings [1–4]. Beyond timely antibiotics and control of the source of infection, the early resuscitation of septic shock focuses on optimizing the delivery of oxygen to vital organs using intravenous (IV) fluid administration, vasopressor infusions, and, occasionally, inotrope infusions [5]. Fluid administration improves organ perfusion up to a point. However, excess IV fluid administration may be associated with increased organ dysfunction and mortality [6–8]. In resource-limited settings, the association between higher volumes of fluid administration and increased mortality appears to be causal [9, 10]. The optimal strategy for the administration of fluid in septic shock in resource-rich settings is unknown.

For approximately 15 years, sepsis resuscitation was dominated by a paradigm termed “early goal-directed therapy” (EGDT) based on the results of a single-center, randomized, “usual care” controlled trial [11]. In that seminal trial, EGDT employed IV fluid administration to target a central venous pressure (CVP) of 8–12 mmHg, vasopressor infusions to target a mean arterial pressure (MAP) > 65 mmHg, and blood transfusions and dobutamine infusions to target a central venous oxygen saturation (ScVO_2_) > 70% using a proprietary catheter to measure ScVO_2_ continuously. However, the benefit of this protocol was not reproduced in three large, international, multicenter trials, albeit in settings in which patients in the “usual care” arms received more aggressive fluid administration than the control arm in the original trial [12–15].

In the aftermath of the “sepsis trilogy” of negative RCTs, clinicians remain uncertain of the optimal method to resuscitate septic shock patients without administering harmful amounts of IV fluid or failing to provide adequate support for organ perfusion. Transthoracic echocardiography (TTE) is a promising technique for guiding hemodynamic management to improve outcomes in septic shock. Echocardiography is non-invasive and available in most contemporary intensive care units (ICUs). Both fluid responsiveness [16–18] and cardiac dysfunction (common in patients with septic shock [19]) can be quickly identified with TTE. The use of echocardiogram-guided IV fluid administration has been shown to improve outcomes in the perioperative period for abdominal, orthopedic, and cardiac surgeries [20–27]. One trial of echocardiogram-guided septic shock resuscitation versus usual care in a pediatric population suggested a shorter time to shock resolution with echocardiogram use [28]. To our knowledge, echocardiogram-guided IV fluid administration in septic shock has not yet been tested in randomized controlled trials in adults.

In a randomized, controlled, feasibility trial, we compared an echocardiogram-guided strategy for the management of septic shock with an EGDT strategy. We hypothesized that echocardiogram-guided management of septic shock would decrease fluid administration and result in more rapid resolution of sepsis-associated organ dysfunction when compared to EGDT.

This study was retrospectively registered at clinicaltrials.gov (identifier NCT02354742, title Echo vs EGDT in severe sepsis and septic shock) on February 3, 2015. Registration was completed before review or analysis of any data.

## Methods

We conducted a single-center, feasibility, randomized controlled trial comparing an echocardiogram-guided fluid and inotrope protocol (ECHO group) to a slightly modified EGDT protocol (EGDT group). The study took place in the Respiratory and Shock-Trauma ICUs at Intermountain Medical Center, a 468-bed academic tertiary care hospital in Murray, Utah.

The target population was adult (≥ 18 years) patients with septic shock and either the presence of or intention to place a central venous catheter and an arterial catheter. Patients were enrolled within 6 h of meeting inclusion criteria. Septic shock was defined according to the second international consensus definition then applicable [29]. We excluded patients who were moribund, pregnant, incarcerated, or for whom immediate surgery was planned. We also excluded patients in whom the protocol could not be performed either due to clinician or patient directives that restricted performing the protocol, or in patients with chest or abdominal pathology that would prevent a limited TTE (e.g., surgical bandages, fresh laparotomy, or left-sided pneumothorax). See Additional file 1: Appendix 1 in the additional digital content for complete inclusion and exclusion criteria.

Patients were screened, consented, and enrolled in the ICU (29 patients) or emergency department (ED, 1 patient) by trained research coordinators. Randomization was performed using random permuted blocks (2, 4, or 6 patients). Allocation was not blinded given the nature of the intervention. Study coordinators were not able to access group allocation until enrollment was complete and randomization performed. The first study assessment was performed as soon as possible on arrival to the ICU. We designed this as an ICU-based study, as the study hospital historically has brief ED length of stay for critically ill patients with sepsis.

**Fig. 1:**
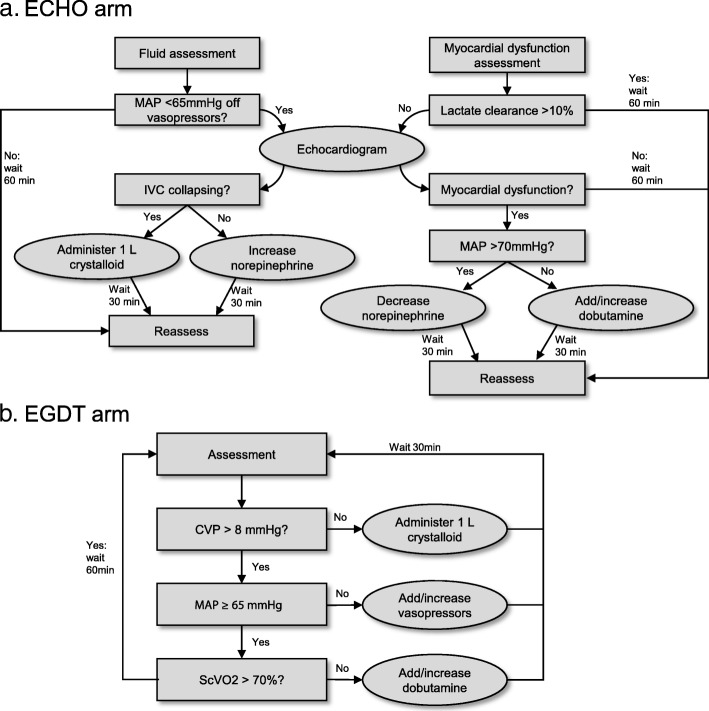
Study protocols for the echocardiography (**a**) and early goal-directed therapy arms (**b**)

The EGDT and ECHO algorithms are depicted in Fig. 1. In both groups, assessments were performed hourly for 6 h. If the algorithm dictated an intervention, an additional assessment was performed 30 min later. In the EGDT group, the assessment consisted of measuring a CVP, MAP, lactate, and ScVO_2_. Fluid was administered in 1 L boluses at each assessment until a CVP of 8–12 mmHg was achieved. If a central line was not yet placed, the shock index (heart rate/systolic blood pressure) was used instead of CVP. Fluid was administered for a shock index ≥ 1 until a central line was placed. When the CVP was at goal, vasopressors were administered to target a MAP ≥ 65 mmHg. Once both CVP and MAP were at goal, ScVO_2_ was checked. If ScVO_2_ was < 70% and lactate clearance was < 10%, dobutamine was initiated at a dose of 5 mcg/kg/min. Dobutamine could be titrated up to a maximum dose of 15 mcg/kg/min to target ScVO_2_ ≥ 70%.

In the ECHO group, each assessment consisted of measuring a lactate and performing a focused TTE to assess the inferior vena cava (IVC) collapsibility and myocardial function. The TTE was either performed by an echocardiographer-physician (testamur of the National Board of Echocardiography’s ASCeXAM with level II training) or performed by a registered diagnostic cardiac sonographer. All images were interpreted by an echocardiographer-physician. Myocardial dysfunction was defined as left ventricular dysfunction (ejection fraction < 45%), right ventricular dysfunction (fractional area change < 35% or tricuspid annular plane systolic excursion < 16 mm), or both. The IVC was assessed for collapsibility and was considered to be collapsing if the maximum diameter was < 5 mm or if vena cava collapsibility index (VCCI), defined as the difference in diameter during a respiratory cycle divided by the maximum diameter, was > 50%. See Additional file 1: Appendix 1 of the additional digital content for a description of VCCI. If the MAP was < 65 mmHg or the patient was on vasopressors and the IVC was collapsing, a 1-L bolus of crystalloid was administered. If the MAP was < 65 mmHg or the patient was receiving vasopressors, and the IVC was not collapsing, vasopressors were increased to target a MAP ≥ 65 mmHg. If vena cava could not be assessed adequately, we substituted a shock index ≥ 1 as an indicator to administer fluid. If systolic dysfunction of either ventricle was identified and the lactate clearance was < 10%, dobutamine was started at a rate of 5 mcg/kg/min if the MAP was < 70 mmHg. If the MAP was ≥ 70 mmHg, the norepinephrine dose was decreased. If lactate clearance continued to be < 10% at the next assessment, dobutamine was up-titrated by 5 mcg/kg/min to a maximum of 15 mcg/kg/min. If the ventricles could not be adequately assessed, dobutamine would not be initiated.

Treatment in both arms included central venous catheter placement, arterial catheter placement, prompt administration of antibiotics, early control of the source of infection, blood transfusions for hematocrit < 21%, consideration of stress-dose steroids for norepinephrine dose > 0.5 mcg/kg/min, and adherence to standard mechanical ventilation guidelines using tidal volumes < 6 ml/kg of ideal body weight.

Process/feasibility endpoints to determine the adequacy of the experimental separation included volume administered during the 6-h study period, volume administered in the first 24 h, and proportion receiving dobutamine at any point during the 6-h study period. The primary clinical endpoint was the change in the Sequential Organ Failure Assessment score (delta SOFA) [30] between day 0 and day 2. Secondary outcomes included 28-day mortality, time to lactate clearance, ICU-free days at 28 days, and ventilator-free days.

Assuming a baseline SOFA score of 8, a sample size of 80 had 80% power (alpha 0.05) to detect a delta SOFA difference of 3 points. Baseline characteristics were described with descriptive statistics, and tests of significance were applied to compare treatment groups. Continuous variables were analyzed using a Mann-Whitney *U* test, and dichotomous variables were compared with the Fisher exact test. Logistic regression was used to adjust mortality for baseline acute physiology and chronic health evaluation, 2nd edition (APACHE II) score [31]. All analyses followed the intention-to-treat principle.

An analysis to assess experimental separation was performed after randomization of 30 patients. When this analysis failed to suggest experimental separation, enrollment was closed.

## Results

**Fig. 2:**
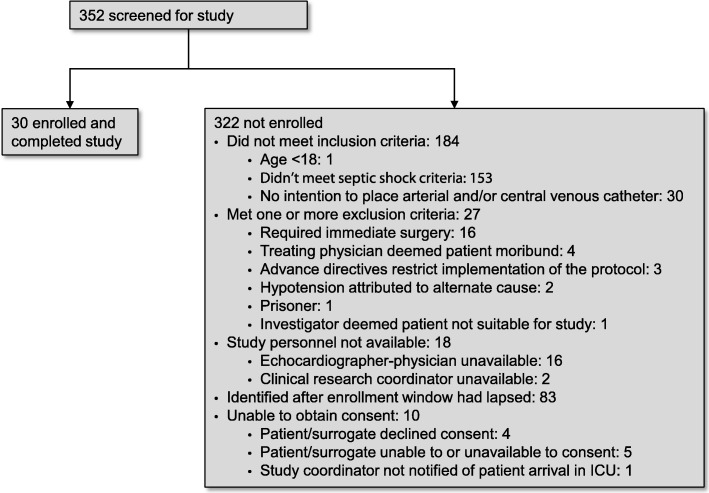
CONSORT diagram describing patient enrollment

Subjects were recruited between January 2015 and May 2017. Three hundred sixty-five subjects were screened for inclusion and 335 were excluded (Fig. 2), primarily for absence of septic shock. Thirty subjects, 15 in each arm, were randomized, completed the trial, and were included in the analysis. Baseline characteristics appeared similar between groups (Table 1).

**Table 1:** Patient characteristics

	ECHO (*n* = 15)	EGDT (*n* = 15)	*p* value
Age (median, IQR)	69 (61,77)	64(49,75)	0.41
Female (*n*, %)	7 (53)	8 (47)	> 0.99
Infection site (*n*, %)			> 0.99
Abdominal	1 (6.7)	2 (13.3)	
Pulmonary	6 (40)	6 (40)	
Skin and soft tissue	2 (13.3)	1 (6.7)	
Urine	4 (26.7)	3 (20)	
Other/unknown	2 (13.3)	3 (20)	
APACHE II (median, IQR)	29 (23,41)	33 (31,41)	0.20
Serum lactate at enrollment, mmol/dL (median, IQR)	3.6 (2.3–5.0)	3.6 (2.0–5.9)	0.97
Mean arterial pressure at enrollment, mmHg (median, IQR)	63 (54–74)	70 (67–84)	0.03
Mechanical ventilation during study period (*n*, %)	2 (13)	6 (40)	0.22
NEE^a^ at enrollment (mcg/kg/min, median, IQR)	0.1 (0,0.29)	0.04 (0, 0.2)	0.45

^a^Norepinephrine equivalent dose

The median time from sepsis identification to randomization was 3.1 h in the ECHO arm and 4 h in the EGDT arm (*p* = 0.33). The median time from sepsis identification to ICU admission was 56 min in the ECHO arm and 82 min in the EGDT arm (*p* = 0.42). Four patients did not meet septic shock criteria until after their ICU admission. Patients in both arms received a median of 3 L crystalloid prior to randomization. This was equivalent to a median of 33 mL/kg in the ECHO group and 38 mL/kg in the EGDT arm (*p* = 0.53, 35 mL/kg for the overall cohort). Every assessment in the ECHO arm was able to adequately characterize the IVC and ventricular function.

There was no significant difference between the ECHO and EGDT arms in the amount of fluid received due to the study protocol (median 0 vs 1 L, *p* = 0.61). The range of fluid administered during the study protocol was 0–7 L in the ECHO arm (mean 1.2 L) and 0–6 L in the EGDT arm (mean 1.4 L). Eleven subjects (73%) in each arm received ≤ 1 L fluid during the study protocol. Total fluid administration in the 24 h after ED arrival was also similar between groups (median ECHO 6.0 vs EGDT 6.4 L, *p* = 0.63).

Three subjects (20%) in the ECHO arm received dobutamine compared to two subjects (13%) in the EGDT arm (*p* > 0.99). Lactate clearance occurred before the first study assessment in 21 patients (11 in the ECHO arm, 10 in the EGDT arm, 70% total study population) and was never elevated in an additional four patients (1 in the ECHO arm and 3 in the EGDT arm, 13% of the total population).

Clinical endpoints appeared similar between the two groups. The median change in SOFA score (a lower value is more favorable) was − 4 in the ECHO arm compared to − 6 in the EGDT arm (*p* = 0.10). Inpatient mortality (5 [33%] vs 3 [20%], *p* = 0.68), APACHE II-adjusted mortality (*p* = 0.18), ICU-free days (median 24.2 vs 24.5 days, *p* = 0.97), and ventilator-free days (median 28 vs 25 days, *p* = 0.51) also appeared similar across groups (Table 2). Overall hospital mortality was 26% across both study groups. No unexpected adverse events or harms were reported in either group.

**Table 2:** Process and clinical outcomes

Process outcomes	ECHO (*n* = 15)	EGDT (*n* = 15)	*p* value
Time from sepsis identification to ICU admission (min)	56	82	0.42
Time from sepsis identification to randomization (h)	3.1	4	0.33
Fluid administration prior to study protocol (median L, median ml/kg)	3, 33	3, 38	0.53
Fluid administration during study protocol (median L [IQR, range])	0 (0, 2, range 0–7)	1 (0, 2, range 0–6)	0.61
24 h fluid administration^a^ (L, median, IQR)	6 (4.7, 8.5)	6.4 (4.8, 9.6)	0.63
Dobutamine administered (*n*, %)	3 (20)	2 (13)	> 0.99
Patients receiving ≤ 1 L of fluid during study protocol (*n*, %)	11 (73)	11 (73)	> 0.99
Clinical outcomes			
Change in SOFA score at 48 h (median, IQR)	− 4 (+ 4 to − 10)	− 6 (− 4 to − 12)	0.10
28-day mortality (*n*, %)	5 (33)	3 (20)	0.68
ICU-free days (median, IQR)	24.2 (0, 25.8)	24.5 (5.4, 25.8)	0.97
Ventilator-free days (median, IQR)	28 (0, 28)	25 (9, 28)	0.51

More protocol instructions were declined by clinicians in the EGDT group than the ECHO group (10% vs 2% of all assessments, *p* = 0.03). Less fluid was administered than was indicated by the study protocol in 50% of those declined protocol instructions (5% of all assessments) in the EGDT group. When the data was analyzed for the median amount of fluid *prescribed by* each protocol (rather than the amount of fluid actually given in each group), there remained no apparent difference between groups (0 L in ECHO vs 1 L in EGDT, *p* = 0.60). Fourteen percent of indicated assessments were missed in the ECHO group compared to 5% in the EGDT group (*p* = 0.08). The most common reason for the missed assessments in the ECHO group was that the echocardiographer-physician was engaged in emergent care of another patient (e.g., providing Advanced Cardiac Life Support or performing an emergent procedure). For a complete list of missed assessments, incomplete assessments, and declined protocol instructions, see Additional file 1: Appendix 2 of the additional digital content.

## Discussion

To our knowledge, no prior randomized controlled trials have investigated the use of echocardiogram-guided resuscitation of septic shock in adults. In this ICU-based randomized controlled feasibility trial of an echocardiogram-guided versus early goal-directed therapy approach to septic shock resuscitation, we did not observe experimental separation between the arms. The lack of experimental separation led to the early completion of this feasibility trial. The target population studied appears relevant to contemporary critical care practice, with an overall hospital mortality of 26%, similar to other recent trials of septic shock [15].

The median time before the first study assessment was only 3.5 h after the patient met criteria for sepsis. Despite prompt identification and enrollment of patients, the probable reason we did not observe experimental separation was that most subjects were adequately volume resuscitated by the time of randomization, with a median of 3 L crystalloid administered before enrollment. The very common achievement of lactate clearance before randomization corroborates this possibility.

The pre-enrollment fluid administration observed in our trial, a median of 3 L or 35 mL/kg, was higher than other recent major trials of early sepsis resuscitation. In the original EGDT study, enrollment depended on hypotension following a fluid bolus of 20–30 mL/kg of crystalloid (or a lactate ≥ 4 mmol/L). However, the amount of fluid administered before initiation of the study protocol is not reported [11]. In the PRISM trial, a patient-level meta-analysis of all patients enrolled in the “sepsis trilogy,” a median of 2 L fluid was administered prior to randomization [15]. Early sepsis management bundles, at 3 and 6 h after sepsis identification, are now mandated by the Centers for Medicaid and Medicare Services (CMS) [32]. A 30 mL/kg fluid bolus is required for patients with septic shock in the 6 h bundle. A retrospective study of almost 50,000 patients in 149 New York hospitals during 2014–2016 showed that the median time to completion of the initial fluid bolus was 2.6 h (IQR 1.3 to 4.2) [33]. Thus, our finding that most patients have received significant fluid resuscitation prior to the median 3.5 h at the first assessment is somewhat higher than recent large trials of sepsis and consistent with recent practice patterns driven by CMS regulatory requirements. Based on the shift in fluid resuscitation practices, trials of early septic shock resuscitation will likely need to focus enrollment exclusively in the ED, before achievement of the CMS bundle targets. Moreover, with more evidence demonstrating the harms of fluid over-administration, it is possible that the target of fluid resuscitation in sepsis may need re-examination [6–10].

More assessments were missed in the ECHO arm than in the EGDT arm in this study. Most of these missed assessments occurred because the echocardiographer-physician, who was frequently the treating physician, was occupied with emergent care of another patient. It is inevitable in the care of ICU patients that the clinician may be engaged in emergent care and not reliably able to perform assessments regularly in each patient with septic shock. The higher number of missed assessments in the ECHO group thus speaks to the relative feasibility of a physician-driven (ECHO) versus nurse-driven (EGDT) assessment. Future trials of echocardiography-directed resuscitation may require training of non-physicians to perform limited echocardiograms. Fortunately, evidence suggests that such personnel could be easily trained for those purposes [34–36]. Although our study demonstrated ability to characterize the IVC and ventricular function in 100% of assessments, these assessments were performed by cardiac sonographers and echocardiographer-physicians. Problems with image acquisition or interpretation might be more prevalent when performed by less well-trained clinicians.

Our choice of threshold for IVC collapsibility was chosen based on published evidence available at the time [37]. The largest study, published after initiation of this study, suggests a lower threshold (25%) may result in more accurate assessment of fluid responsiveness [18]. The consensus criteria for defining sepsis and septic shock have been updated since the initiation of this study [38], so our findings may not be generalizable to patients identified using the newer definition. All patients in our study would have met the new sepsis-3 criteria, but four (2 in each group) would not have met criteria for septic shock given an initial lactate < 2 mmol/L despite receipt of vasopressor infusions. Last, ventilator status may affect both CVP and IVC size and dynamics.

This trial specifically addressed the use of echocardiography to guide resuscitation in early septic shock. The application of focused critical care echocardiography to undifferentiated shock is a separate and important area of study. Several studies of ICU patients have shown that the use of echocardiography, often combined with other point-of-care ultrasound exams, frequently changes the diagnosis and management plan in a broad spectrum of ICU patients, including undifferentiated shock [39–41]. These management changes often included fluid and inotrope modulation based on ultrasound findings. While there is some overlap of our patient population with those studies, our findings cannot be generalized to patients with undifferentiated shock.

The primary limitation of our study is the small sample size. Our study is underpowered to detect a change in clinical endpoints and therefore should not be used to verify non-inferiority of echocardiography versus EGDT. However, given the lack of experimental separation, we believe it is unlikely that a larger sample size with the present trial design would have provided reliable evidence of efficacy. Given the nature of the interventions, blinding (e.g., through sham echocardiography and complete separation of protocol instructions from clinical care) was not feasible, which is another limitation of the trial. There were significantly more clinician-declined protocol instructions, predominantly resulting in less fluid administration in the EGDT group. This suggests that aspects of that protocol made clinicians hesitant to administer the prescribed amount of fluid. Early lactate clearance has been shown to predict improved outcomes in septic shock [42]. However, given the rapid lactate clearance, we observed in this population prior to enrollment that metric may be infeasible in many practice environments.

## Conclusions

Despite evidence in the perioperative period for echocardiogram-guided hemodynamic management, in our ICU-based feasibility trial in patients with early septic shock, we did not observe experimental separation. This lack of experimental separation likely derives from the fact that most patients have received substantial fluid before ICU admission. We believe that further randomized trials of echocardiography-guided resuscitation in septic shock are indicated but will likely require intervention in an even earlier resuscitation window shortly after the patient’s arrival in the ED.

## Additional file

Additional file 1: **eTable S1.** Inclusion and exclusion criteria. Description of vena cava collapsibility index. **eTable S2.** Missed assessments, incomplete assessments, and declined protocol instructions. (DOCX 19 kb)
