# Supplementary material for: Echocardiogram-guided resuscitation versus early goal-directed therapy in the treatment of septic shock: a randomized, controlled, feasibility trial
Source: J Intensive Care. 2018 Aug 13;6:50. doi: 10.1186/s40560-018-0319-3 (PMC6090604; doi:10.1186/s40560-018-0319-3)
Supplement: Supplementary file 1 — eTable S1. Inclusion and exclusion criteria. Description of vena cava collapsibility index. eTable S2. Missed assessments, incomplete assessments, and declined protocol instructions. (DOCX 19 kb) [file 40560_2018_319_MOESM1_ESM.docx]

SUPPLEMENTAL DIGITAL CONTENT

**A randomized, controlled, feasibility trial of echocardiogram-guided resuscitation versus early goal-directed therapy in the treatment of septic shock**

Michael J. Lanspa, MD, MS, FASE, FCCM^1,2,3^, Rebecca E. Burk, MD^1,2,3^, Emily L. Wilson, MS^1^, Eliotte L. Hirshberg, MD, MS, FCCM^1,2,3,4^, Colin K. Grissom, MD, FASE, FCCM^1,2,3^, Samuel M. Brown, MD, MS, FASE, FCCM^1,2,3^

1. Division of Pulmonary and Critical Care Medicine, Intermountain Medical Center, Murray, UT 84107, USA
2. Critical Care Echocardiography Service, Intermountain Medical Center, Murray, UT 84107, USA
3. Division of Pulmonary and Critical Care Medicine, University of Utah School of Medicine, Salt Lake City, UT 84132, USA
4. Division of Critical Care, Department of Pediatrics, University of Utah School of Medicine, Salt Lake City, UT 84132, USA

**Appendix 1. Supplemental methods 2**

eTable 1: Inclusion and exclusion criteria 2

Description of vena cava collapsibility index 2

**Appendix 2. Supplemental results 3**

eTable 2: Missed assessments, incomplete assessments and declined protocol 3

instructions

**APPENDIX 1: Supplemental methods**

eTable 1. Inclusion and exclusion criteria

| Eligibility criteria for study enrollment | |
| --- | --- |
| Inclusion criteria | |
| 1. Septic shock  - ≥2 SIRS criteria^a^ - Suspected or confirmed infection - Evidence of refractory hypotension^b^ | |
| 1. Intention to place an arterial and central venous catheter | |
|  | |
| Exclusion criteria | Rationale for exclusion |
| Age <18 | Children are physiologically distinct from adult patients |
| Pregnancy | Ethical considerations |
| Requirement for immediate surgery | Protocol not possible in the operating room |
| Hypotension attributable to another cause | Shock not associated with sepsis is a distinct physiologic state |
| Patient deemed moribund or aggressive care deemed inappropriate by treating physician | Moribund status profoundly limits possible effects of treatment |
| Advanced directives restrict aspects of the protocol | Ethical considerations |
| Contraindication to central venous catheter or arterial catheter placement | Inability to perform aspects of both study arms |
| Chest or abdominal wall pathology restricting ability to perform echocardiography | Inability to perform the ECHO arm protocol |

a. Systemic inflammatory response syndrome (SIRS) criteria are white blood cell count <4,000/mm^3^ or >12,000/mm^3^ or >10% immature forms, heart rate >90 beats/minute, respiratory rate >20 breaths/minute or PaCO_2_ <32mmHg, and temperature <36°C or >38°C

b. Defined as a systolic blood pressure <90mmHg despite a 30mL/kg fluid challenge or a serum lactate > 4mmol/L

**Description of vena cava collapsibility index:**

VCCI = (IVC_max_ - IVC_min_) / IVC_max_

VCCI: vena cava collapsibility index

IVC_max_: Maximum diameter of the IVC during the respiratory cycle

IVC_min_: Minimum diameter of the IVC during the respiratory cycle

**APPENDIX 2: Supplemental results**

eTable 2. Missed assessments, incomplete assessments and declined protocol instructions

|  | EGDT | ECHO |
| --- | --- | --- |
| Total indicated assessments* (n) | 131 | 143 |
| **Missed assessments** (n)** |  |  |
| Patient not in ICU, e.g. at an imaging study | 2 | 0 |
| Echo physician providing emergent care to another patient | 0 | 12 |
| Patient undergoing procedure | 1 | 1 |
| Prior intervention incomplete | 0 | 3 |
| Other | 4 | 2 |
| **Incomplete assessments*** (n)** |  |  |
| Labs not resulted | 3 | 0 |
| Patient undergoing procedure | 3 | 0 |
| **Declined protocol instructions**** (n)** |  |  |
| Provider did not want additional fluid administered | 7 | 1 |
| Provider did not want dobutamine administered | 2 | 0 |
| Provider chose lower amount of fluid than protocol dictates | 2 | 0 |
| Provider chose albumin over crystalloid | 3 | 0 |
| Provider awaiting other diagnostic imaging before administering fluid | 0 | 1 |

***Total indicated assessments-**The total number of assessments that were indicated for all the subjects in each group. A minimum of 6 hourly assessments were indicated for each patient. Additional assessments were indicated at 30 minutes if an intervention was performed. Thus, the total number of indicated assessments in each group was dependent on the number of interventions performed.

****Missed assessments-**The assessment was not completed

*****Incomplete assessments-**The assessment was attempted, but not all information was available in order to complete the assessment

******Declined protocol instructions-**The assessment was completed, but the clinical attending declined the protocol instruction
